# Effect of resilient architecture in an ancient windmill in the Sistan region on natural ventilation enhancement

**DOI:** 10.1038/s41598-022-23027-w

**Published:** 2022-10-29

**Authors:** Elham Mohammadi, Mohammadreza Jarkeh, Seyed Alireza Zolfaghari, Vahid Arbabi

**Affiliations:** 1 Tose Sazan Pahne Kiyansea (TSPK) Company, Incubator Center, University of Zabol, Zabol, Iran; 2 Department of Mechanical Engineering, Faculty of Engineering, University of Birjand, Birjand, Iran; 3 Department of Orthopedics, University Medical Center Utrecht, Utrecht, The Netherlands

**Keywords:** Engineering, Civil engineering, Mechanical engineering, Energy science and technology, Energy harvesting, Renewable energy

## Abstract

Over centuries different elements have been developed in architectures for ensuring adequate natural ventilation in residential units. This study assesses the different components of an ancient windmill in Sistan, Iran, on the structure's indoor air quality (IAQ) enhancement. Several climatic scenarios have been defined by the wind analysis of Sistan meteorological data and analyzed by CFD. The site measurements confirm the accuracy of the simulation results. In the windmill, two deflectors facing the prevailing wind are the significant elements which, in addition to directing wind toward the entrance, could form vortices near the east and west openings leading to suction ventilation. Alteration of the wind speed and angle from 10 to 15 m/s and 30° to 17° would increase the air change per hour (ACH) by 150% and 110%, respectively. Meanwhile, the ACHs were higher than the ASHRAE desired level (ACH > 0.35).

## Introduction

Nowadays, occupants' well-being in buildings places a strain on energy by increasing fossil fuel consumption. Zero net energy (ZNE) architecture would be a solution to reducing energy use. In ZNE, the natural ventilation^1–6^ in dwellings has long been common in arid^7^ and windy areas, such as some parts of Iran. Residents of these areas have utilized wind in and around buildings for centuries to improve indoor air quality without consuming energy. An examination of these works shows that they were undoubtedly aware of all aerodynamics principles and applied them in their designs.

The design parameters of openings and additional ventilating tools play an essential role in wind-induced ventilation performance. The effect of type and positions of transom windows^8^ (TWs) on ACH has been evaluated in high-rise buildings^1^. A combination of CFD analysis and the artificial neural network has indicated that the ACH would increase to 108.1% on average by proper design of TWs (the dimension and direction of windows in accordance with the velocity and direction of the wind).

Wind towers, also known as wind-catcher, Kolak et al.^9^, are typically vertical structures built on the roof of buildings with mainly rectangular openings facing the prevailing wind for cooling indoors^10,11^. Site measurements^12–15^ as well as numerical studies^9,16–22^ confirm the effectiveness and reliability of these structures, introducing them as new solutions to ventilate modern buildings naturally^23–26^.

Wing walls or deflectors^27^ are vertical^28^ or horizontal^29^ solid elements around and near windows or windcatchers^30^, enhancing natural single-side ventilation^31,32^ by creating extra pressure gradients among openings. It can induce natural suction ventilation by producing a low-pressure region on outlet windows^33^. This element is efficient when the wind flows by an oblique angle, and the performance diminishes if the wind direction tends to 90°^32^. Although the wing wall has been used in vernacular buildings (casement windows pivoting outward), modern architectures utilize its practical application in modern design; for example, the UMNO office corridors can be naturally ventilated by its wing walls^34,35^. The use of wing walls on the edge of a windcatcher revealed that this element would supply a maximum of 9.6 kW cooling capacity at 4 m/s wind speed, which could help to maintain the indoor temperature below the maximin adaptive temperature^30^.

The indoor air quality would diminish if the outside air is warm^36^, warm-humid, and polluted^37^. So, alternative methods are suggested to prevent direct outdoor–indoor air interaction under intensely hot/cold outdoor conditions. Concerning warm outdoor conditions, the combination of evaporation cooling and the solar chimney was suggested^7,38–41^ to decrease the indoor temperature by about 8 °C when the outdoor temperature was higher than 35 °C. In addition, as the applicability of evaporation cooling is not recommended in warm-humid regions, the venturi-shape windcatcher^42^ was designed which can enhance the ventilation rate by 8 times more than cross-ventilation^43^. This structure was designed based on the Bernoulli effect^44^ which explains how the negative pressure occurs when the speed increases on one side of the venturi-shape structure leading to suction-ventilation. Further, in air polluted conditions, the outdoors can affect negatively IAQ^7,45,46^ and the use of filters on entrances was recommended^47^. However, few studies have been performed on the couple effects of filters with windcatchers and solar chimneys as an effective way of inducing ventilation^7,47^.

Environmental pollution in Sistan (a region on the border of Iran and Afghanistan), where dust storms^48–50^ occasionally occur because of dry conditions and 120-day winds^51^, introduced this region as the most polluted city in 2016^52^. In this region, ancient architectures utilized wind which, in addition to cooling, could control dust entrance to buildings^15,19,21^. Heidari has identified three elements regarding cross-ventilation in Sistan's buildings^19^, i.e., a windcatcher (called "Kolak"), openings (called "Daricheh"), and a wall groove^28^ (called "Surak"). Davtalab^53^ has identified a Sistani traditional element, an inlet opening called Kharkhona, which can affect human comfort by simultaneously utilizing wind and humidity in arid areas^53^. The results indicated that Kharkhona would transfer the indoors from "very hot" and "hot" to "warm" conditions by reducing Physical Equivalent Temperature (PET) and elevating humidity by 9 °C and 30%, respectively.

This study applied CFD analyses to assess the wind-driven ventilation mechanism employed in an ancient windmill in the Hozdar region, Sistan, Iran, at a longitude of $61.27^\circ$ and latitude of $30.55^\circ$. The buoyancy effect causes air change in the building due to the temperature difference between indoors and outdoors. However, in this study, the buoyancy ventilation was considered negligible since, in arid areas, the temperature gradient between indoors and outdoors is not significant^6,43,54^. Long-term meteorological data were analyzed to assess the wind speeds and directions considered for four scenarios of the CFD analyses, i.e., two different wind angles by two different wind speeds.

The windmill consists of three internal parts; the workplace (where occupants spent most of their time), the entrance, and the rear. The structure was orientated toward prevailing wind with two vertical deflectors constructed at the specified angles and lengths on the windward. Other ventilation elements included one opening as an inlet, six openings on two sidewalls (four out of six were located in two corridors built on two sidewalls of the workplace, and two remaining openings on sidewalls of the entrance), and two openings on the roof.

This paper aimed to assess how the ancients used elements in their architecture to enhance indoor air quality at a desirable level by natural ventilation. Indeed, the IAQ can be investigated by different criteria; in this research, the ACH was introduced as a well-known criterion based on international standards. The present study investigated how deflectors can cause the formation of vortices behind them and near the sidewalls, leading to natural suction ventilation in a wide range of incident angles.

## Methodology

### Statistical analysis of wind in Sistan

**Figure 1:**
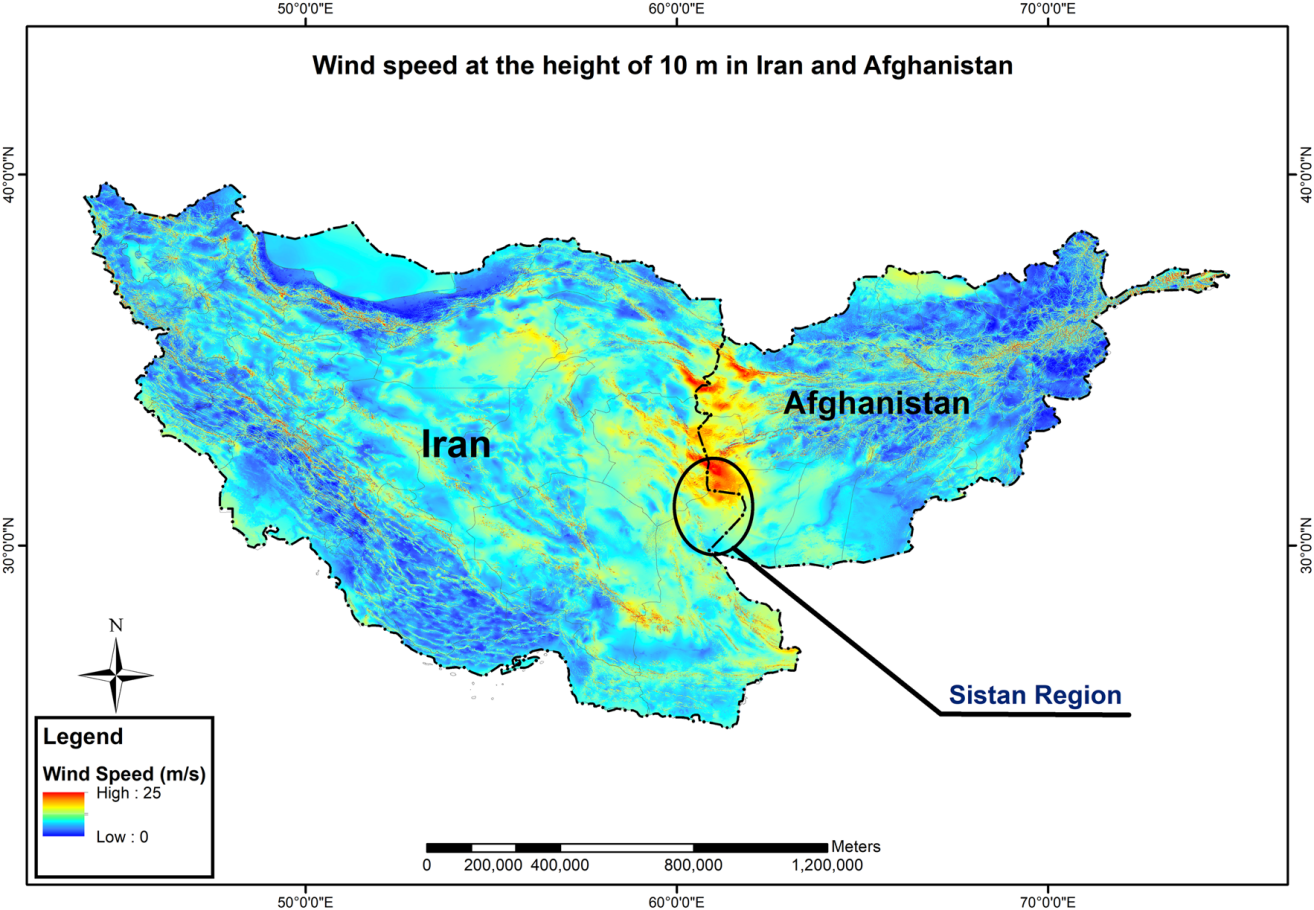
The highest wind speed occurring in the Sistan region^62^.

Sistan is a region located in the eastern part of Iran and the western part of Afghanistan. Investigations about wind energy in Iran^55–61^ and the global wind atlas^62^ indicated that Sistan is a region with the highest wind energy potential in Iranian territory (Fig. 1).

**Figure 2:**
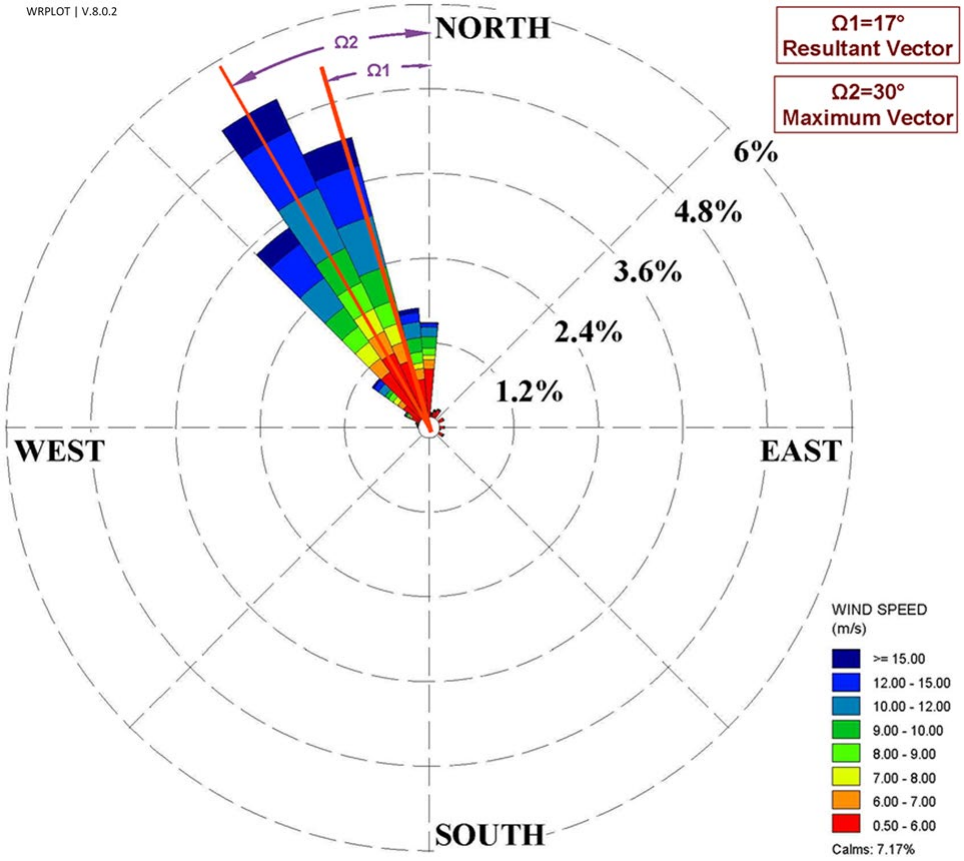
The wind rose of Sistan, as well as two dominant wind directions.

The 56-year (from February 1963 to February 2020) wind data at Zabol airport meteorological station (Station ID = 40,829), located at 31° 5′ N longitude and 61° 32′ E latitude was used for wind statistical analyses. The wind speed and direction distribution have been analyzed and shown in the wind rose. The results indicated that the dominant wind directions were from northwest to southeast from $315^\circ$ to $360^\circ$. The angle of the resultant vector was $343^\circ$ (or $17^\circ$ counterclockwise from the North direction, Ω1), and the dominant wind direction was $330^\circ$ (or $30^\circ$ counterclockwise from the North direction, Ω2) (Fig. 2).

**Figure 3:**
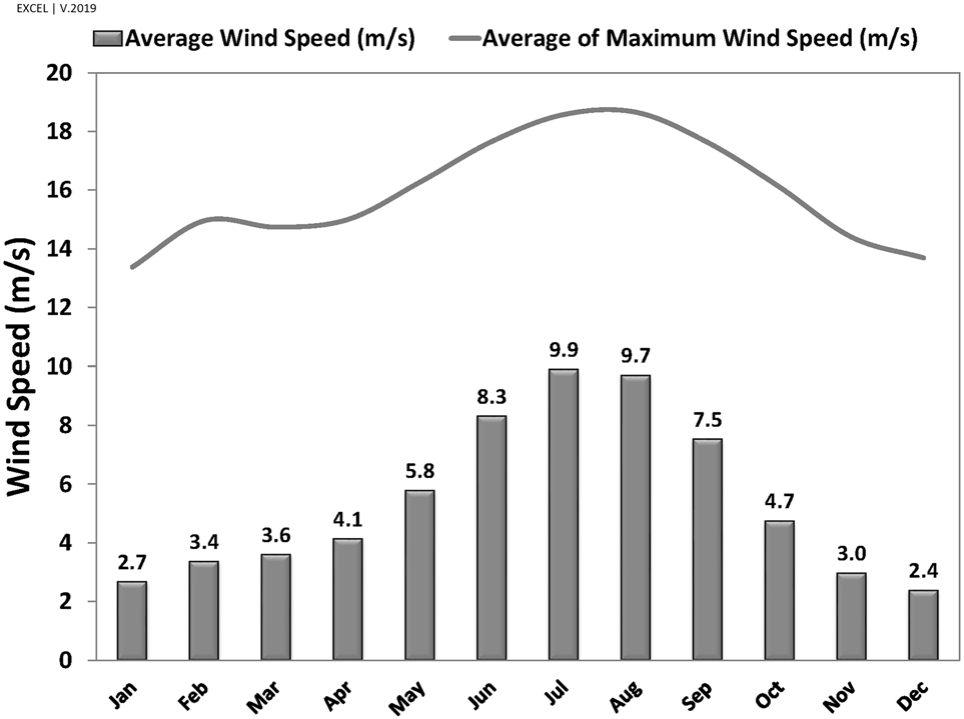
Average and Average of maximum wind speed (1963–2020).

In addition, the analyses reveal that although the average wind speed increased to near 10 m/s (or 36 $\mathrm{km}/\mathrm{h}$) in July, the annual average of top wind speeds is approximately 15 m/s (or 54 $\mathrm{km}/\mathrm{h}$) (Fig. 3).

### Wind-driven ventilation in the windmill

#### Geometry and boundary conditions

**Figure 4:**
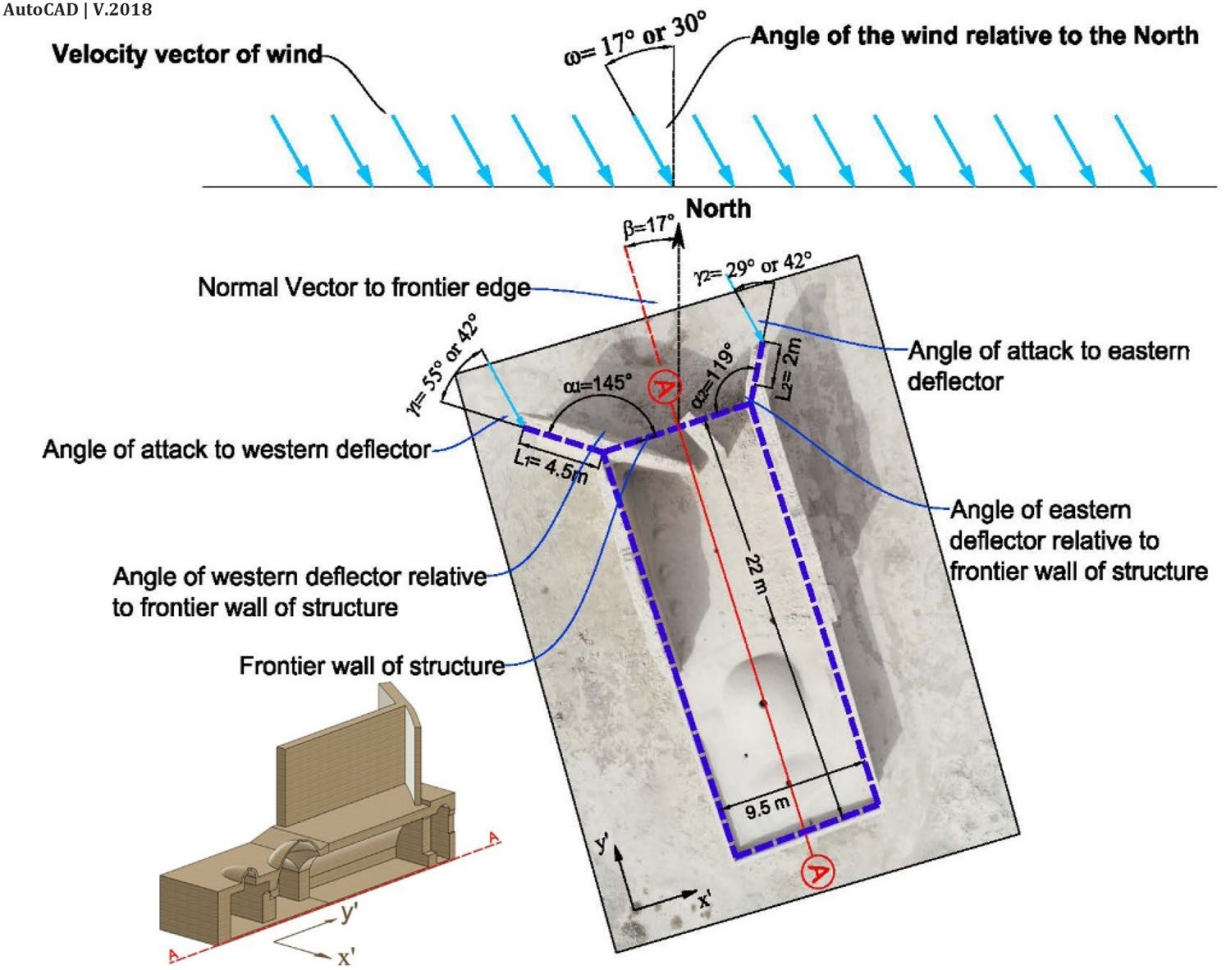
Dimension of elements in the windmill.

Significant angles of the windmill are depicted in Fig. 4. Two deflectors were constructed on the structure's frontier wall to confront the prevailing wind. The deflectors had two main functions:

- The most important function was to accumulate and direct the wind toward the propeller^63–69^ and wind entrance.
- The second function was to reduce the turbulent flow (by forming vortices) on the east and west wall for enhancing indoor ventilation.

The west deflector was constructed by the angle of ${\alpha }_{1}=145^\circ$ and the length of ${L}_{1}=4.5 \mathrm{m}$, where the angle of the east deflector was ${\alpha }_{2}=119^\circ$ with the length of ${L}_{2}=4 \mathrm{m}$.

**Figure 5:**
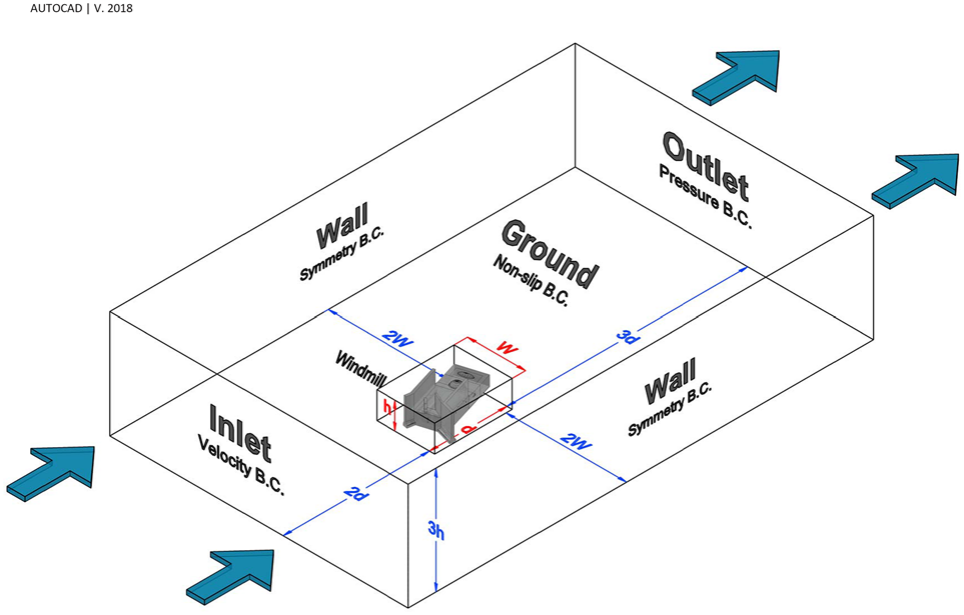
Domain dimensions and boundary conditions.

According to the deflectors and the angle of structure ($17^\circ$ in the NE-SW direction), the dimensions of the building block in this windmill were 9.5 m (W)$\times$ 22 m(length) $\times$ 9 m(height), (Fig. 5). The CFD domain was allocated according to Autodesk guidance^70^. The dimensions of the domain were 5 W (97.5 m) $\times$ 6d (156 m) $\times$ 3 h (27 m). Regarding CFD analysis, four different boundary conditions were allocated to the domain: the steady-state velocity condition for the inlet (different for each scenario), slip/symmetry condition for lateral and top boundaries, zero pressure condition for the outlet, and non-slip condition for the ground.

**Figure 6:**
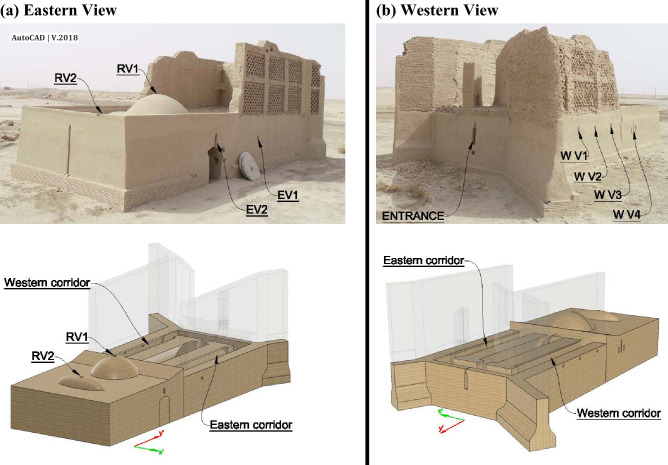
Elements on (**a**) eastern and (**b**) western walls of the windmill.

In this windmill, nine openings have been considered for natural ventilation. Four vents have been embedded in two corridors (which were constructed for preventing indoor turbulent flow), i.e., three western corridor vents (annotated by WV1, WV2, and WV3) and one eastern corridor vent (annotated by EV1) (Fig. 6). Further, three vents were constructed in the middle room, one on the western side, WV4, one above the door as a transom windows^1^, EV2, and one on the roof acting as the chimney, RV1. The vent RV2 was an identical chimney on the roof of the rear room. A vent on the frontier wall (ENTRANCE) served as an air intake.

#### Scenarios

**Figure 7:**
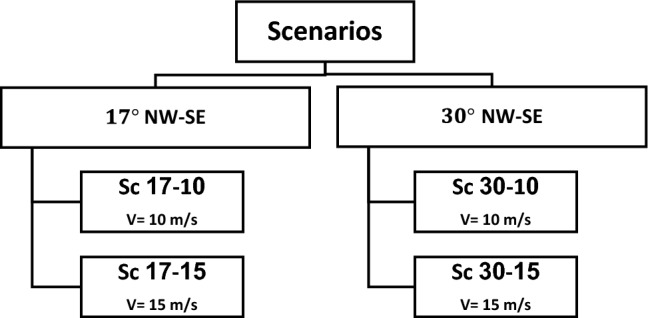
Scenarios for CFD of wind-driven ventilation.

According to wind rose in Sistan (Fig. 2), the resultant wind vector and dominant wind vector are $\Omega 1=17^\circ$ and $\Omega 2=30^\circ$, respectively. Further, the maximum monthly wind speed (in July) and an average maximum wind speed are 10 m/s and 18 m/s (Fig. 3). Thus, this study considered four different scenarios based on a statistical analysis of wind in Sistan: two angles of $17^\circ$ (the angle of resultant wind vector as well as the windmill) and 30°, and two different wind speeds, 10 $\mathrm{m}/\mathrm{s}$ and 15 $\mathrm{m}/\mathrm{s}$ (Fig. 7).

#### Governing equations

The pseudo-steady-state incompressible Reynolds-average Navier–stokes (RANS) for the two-equation $k-\varepsilon$ turbulent model^30^ has been chosen for considering the model accuracy and computational cost^71^. In other words, the ventilation model was carried out only by considering the wind. The governing equations have been listed in Eqs. (1)–(5)^4,70^.

Continuity:

1 $$\frac{\partial \overline{u}{ }_{i}}{\partial x{}_{i}}=0$$

Momentum conservation:

2 $$\frac{\partial \overline{{u }_{i}{u}_{j}}}{\partial x{}_{i}}=-\frac{1}{\rho }\frac{\partial \overline{p}}{\partial x{ }_{i}}+\frac{\partial }{\partial x{}_{j}}\left(\vartheta \left(\frac{\partial \overline{u}{ }_{i}}{\partial {x}_{j}}+\frac{\partial \overline{u}{ }_{i}}{\partial x{}_{i}}\right)\right)$$

The Eddy viscosity (${v}_{t}$) is calculated in terms of $k$ and $\varepsilon$ by

3 $${\vartheta }_{t}={C}_{\mu }\frac{{k}^{2}}{\varepsilon }$$

Turbulent kinetic energy (TKE):

4 $$\frac{\partial k\overline{u}{ }_{i}}{\partial {x}_{i}}=\frac{\partial }{\partial x{}_{i}}\left(\frac{{\vartheta }_{t}}{{\sigma }_{k}}\frac{\partial k}{\partial {x}_{i}}\right)+\vartheta \left(\frac{\partial \overline{u}{ }_{i}}{\partial {x}_{j}}+\frac{\partial \overline{u}{ }_{j}}{\partial x{}_{i}}\right)\frac{\partial \overline{u}{ }_{j}}{\partial x{}_{i}}-\varepsilon $$

Turbulent dissipation rate (TKE):

5 $$\frac{\partial \varepsilon \overline{u}{ }_{i}}{\partial {x}_{i}}=\frac{\partial }{\partial x{}_{i}}\left(\frac{{\vartheta }_{t}}{{\sigma }_{k}}\frac{\partial k}{\partial {x}_{i}}\right)+{C}_{1\varepsilon }\frac{\varepsilon }{k}\vartheta \left(\frac{\partial \overline{u}{ }_{i}}{\partial {x}_{j}}+\frac{\partial \overline{u}{ }_{j}}{\partial x{}_{i}}\right)\frac{\partial \overline{u}{ }_{j}}{\partial x{}_{i}}-{C}_{2\varepsilon }\frac{{\varepsilon }^{2}}{k}$$

where $\overline{u}{ }_{i}$ and $\overline{p }$ are the averaged velocity components and pressure, respectively. The five modeling constants ${C}_{1\varepsilon }$, ${C}_{2\varepsilon }$, ${C}_{\mu }$, ${\sigma }_{k}$, and ${\sigma }_{k\varepsilon }$ are 1.44, 1.92, 0.09, 1.0, and 1.3, respectively.

### Model validation

**Figure 8:**
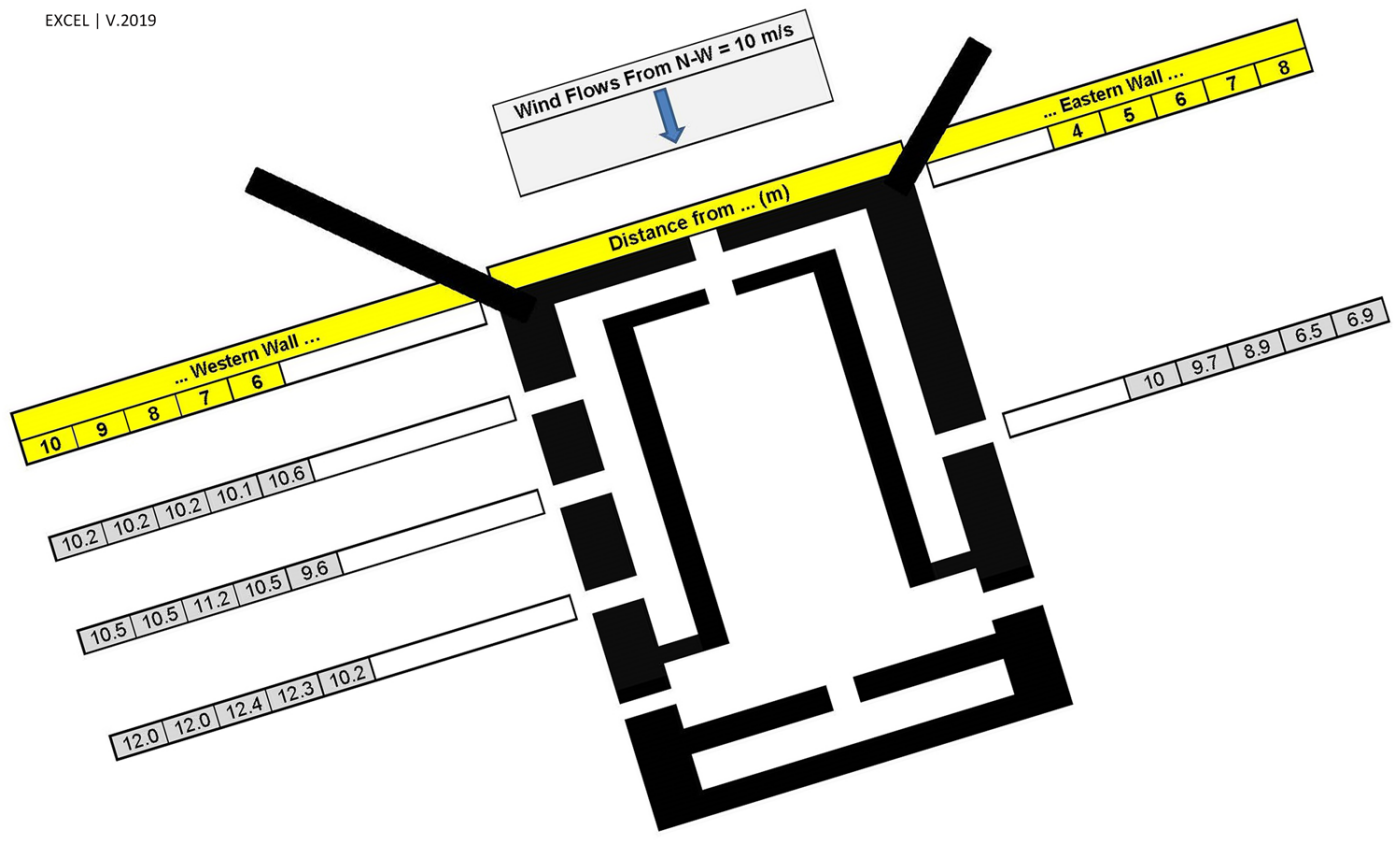
Field measurement of wind velocity on profiles perpendicular to vents.

Wind direction and speed across three western and eastern corridor vents were measured by an anemometer in different sections near the walls. The anemometer used in this study was UNI-T UT363 with wind speed accuracy and resolution of (± 5%rdg + 0.5 m/s) and 0.1 m/s, respectively. The measurements were done in May 2020, where the average wind speed and direction were 10 m/s and $20^\circ$, respectively (Fig. 8). The wind speed at specified locations was measured while the wind condition stayed identical around the windmill during measurement.

The root means square error, RMSE, (Eq. 6) value was used to evaluate the validation of the CFD results.

6 $$RMSE={\left(\frac{1}{n}\sum_{i=1}^{n}{\left({U}_{o}^{i}-{U}_{s}^{i}\right)}^{2}\right)}^{1/2}$$

where ${U}_{o}^{i}=({V}_{o}^{i}/{V}^{\infty })$ is the dimensionless measured average velocity in position *i* and ${U}_{s}^{i}=({V}_{s}^{i}/{V}^{\infty })$ represents the dimensionless simulated average velocity in the same position, and *n* denotes the number of measurements. The reference velocity in the measurement date was about ${V}^{\infty }=10$ m/s. According to the mentioned parameter, the average margin error of 17% was obtained (Table 1).

**Table 1:** Validation in the windmill model.

Vent no.	Distance (m)	Measure velocity (m/s)	Simulated velocity (m/s)	${U}_{o}^{i}$	${U}_{s}^{i}$	${\left({{U}_{o}^{i}-U}_{s}^{i}\right)}^{2}$
W1	6	10.6	7.5	1.06	0.75	0.0956
W1	7	10.1	8.1	1.01	0.81	0.0386
W1	8	10.2	10.9	1.02	1.09	0.0046
W1	9	10.2	11.2	1.02	1.12	0.0092
W1	10	10.2	11.3	1.02	1.13	0.0127
W2	6	9.6	8.9	0.96	0.89	0.0043
W2	7	10.5	9.7	1.05	0.97	0.0067
W2	8	11.2	11.0	1.12	1.10	0.0004
W2	9	10.5	11.2	1.05	1.12	0.0047
W2	10	10.5	11.2	1.05	1.12	0.0054
W3	6	10.2	8.8	1.02	0.88	0.0190
W3	7	12.3	9.0	1.23	0.90	0.1122
W3	8	12.4	10.5	1.24	1.05	0.0376
W3	9	12.0	11.0	1.20	1.10	0.0100
W3	10	12.0	11.1	1.20	1.11	0.0089
E1	4	10.0	8.5	1.00	0.85	0.0227
E1	5	9.7	10.0	0.97	1.00	0.0010
E1	6	8.9	10.7	0.89	1.07	0.0330
E1	7	6.5	11.1	0.65	1.11	0.2086
E1	8	6.9	11.0	0.69	1.10	0.1650
$RMSE={\left(\frac{1}{n}\sum_{i=1}^{n}{\left({U}_{o}^{i}-{U}_{s}^{i}\right)}^{2}\right)}^{1/2}$						17%

### Grid independence analysis

**Figure 9:**
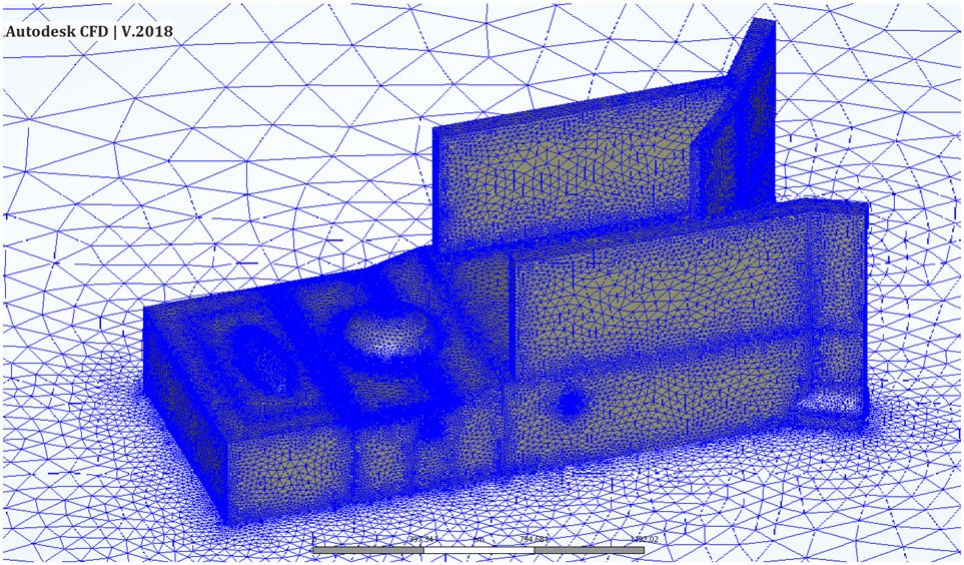
Schematic view of the CFD mesh.

The unstructured tetrahedral mesh was generated by Autodesk CFD to perform the simulation. The computational domain was composed of ~ 5.6 million cells as the reference mesh (Fig. 9). Because of the complexity of geometry, the computational domain was refined in critical zones such as the vents and corridors.

Grid independence analysis was conducted to ensure that fining the grid resolution would not significantly affect the results. Thus, coarse, medium, and fine meshes were generated for Sc17-10 in the CFD model. The average difference, ${R}_{a}$ (Eq. 7), between the velocity profiles for different mesh sizes was defined to study the mesh independence criteria.

7 $${R}_{a}=100\times \frac{1}{n}\sum_{i=1}^{n}\frac{\left|{V}^{*}-V\right|}{{V}^{*}}$$

where ${V}^{*}$ is the velocity value at finer mesh rather than $V$ for coarser mesh. Table 2 presents the total number of cells and the percentage difference between the velocity profiles over five different lines being perpendicular to the vents EV1, WV1, WV2, WV3, and WV4, respectively. As seen in Table 1, the ${R}_{a}$ between the coarse and medium grid is close to 1.5% and between the medium and fine mesh is below 1%. Thus, the mesh with about 5.8 million cells was chosen as the main mesh for the present study.

**Table 2:** Grid independence analysis.

No.	Mesh type	Cell counts	${R}_{a}$ (%)					Mean ${R}_{a}$ (%)
			EV1	WV1	WV2	WV3	WV4	
1	Coarse	4,596,275	1.14	0.57	0.76	1.34	3.40	1.44
2	Medium	5,608,715	0.68	0.25	0.20	0.49	0.82	0.49
3	Fine	6,845,296						

### Solution convergence

To monitor the convergence, the automatic convergence assessment^71^ was selected. At first, the criteria were set to moderate, while the accuracy was increased by changing the criteria to tight. Also, the following solution convergence metrics were calculated during the convergence check:

1. Instantaneous Convergence Slope: This metric calculated the slopes of the convergence data from one iteration to the next. When the maximum instantaneous slope was below the set level, the solution was stopped. In the study, the set value was defined as $1\times {10}^{-4}$.
2. Time Averaged Convergence Slope: This metric evaluated the slope of the convergence data over several iterations. The threshold value was set on $1\times {10}^{-2}$.
3. Time Averaged Convergence Concavity: The derivation of maximum time-averaged convergence slope is a measure of whether the curve is flattening (slope is descending) or growing (slope is ascending). The solution will stop when the concavity is less than the set value. This value was set on $1\times {10}^{-2}$ in tight criteria.
4. Field Variable Fluctuations: It calculates the standard deviation of the dependent variable where the solution will stop when the deviation is below the set level. This value was set on $1\times {10}^{-5}$ in this study.

## Results

In this study, CFD analyses have revealed vortices' performance behind deflectors and assessed how they affect indoor wind-driven ventilation. The ventilation was based on the wind current in a boundary layer near the vents, which would lead to pressure drop and vacuum in them.

### Impact of deflectors on ventilation

**Figure 10:**
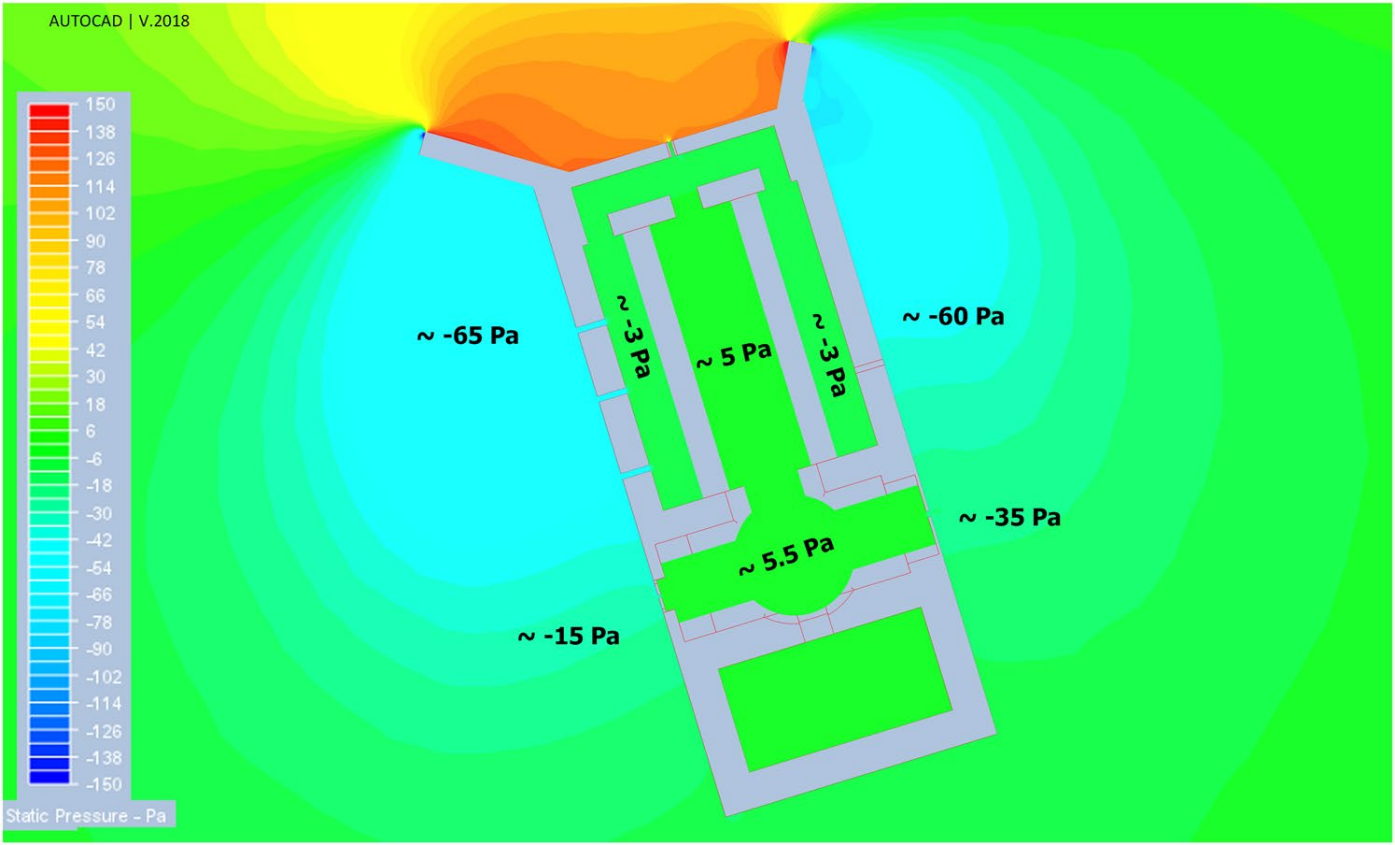
Pressure distribution around and inside the windmill.

**Figure 11:**
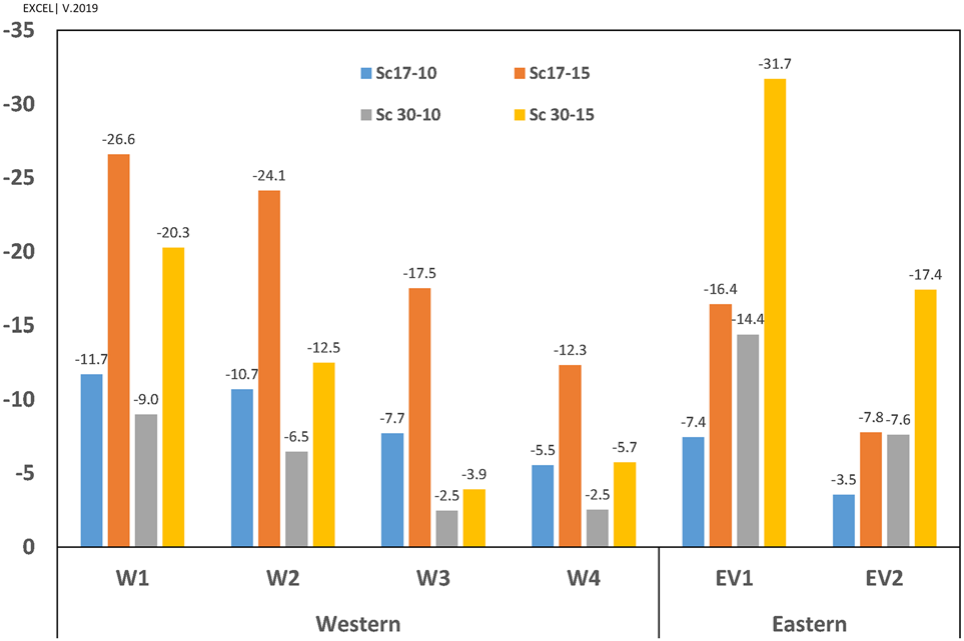
Pressure gradient in four scenarios for western and eastern vents.

In this structure, deflectors play a crucial role in natural ventilation. They can provide air circulation due to the induced pressure gradient between the interior and exterior of the building. Thus, to understand the necessity of deflectors, the model’s total pressure distribution is presented in Fig. 10. As depicted in Fig. 10, the circulation on the leeward of the deflectors provides a negative pressure gradient (the outdoor pressure is higher than indoor vents). The pressure gradient and air velocity through the vents in four scenarios have been shown in Fig. 11. This led to suction forces that ventilated the indoor air through corridor vents as well as WV4 and EV2.

### Flow pattern

**Figure 12:**
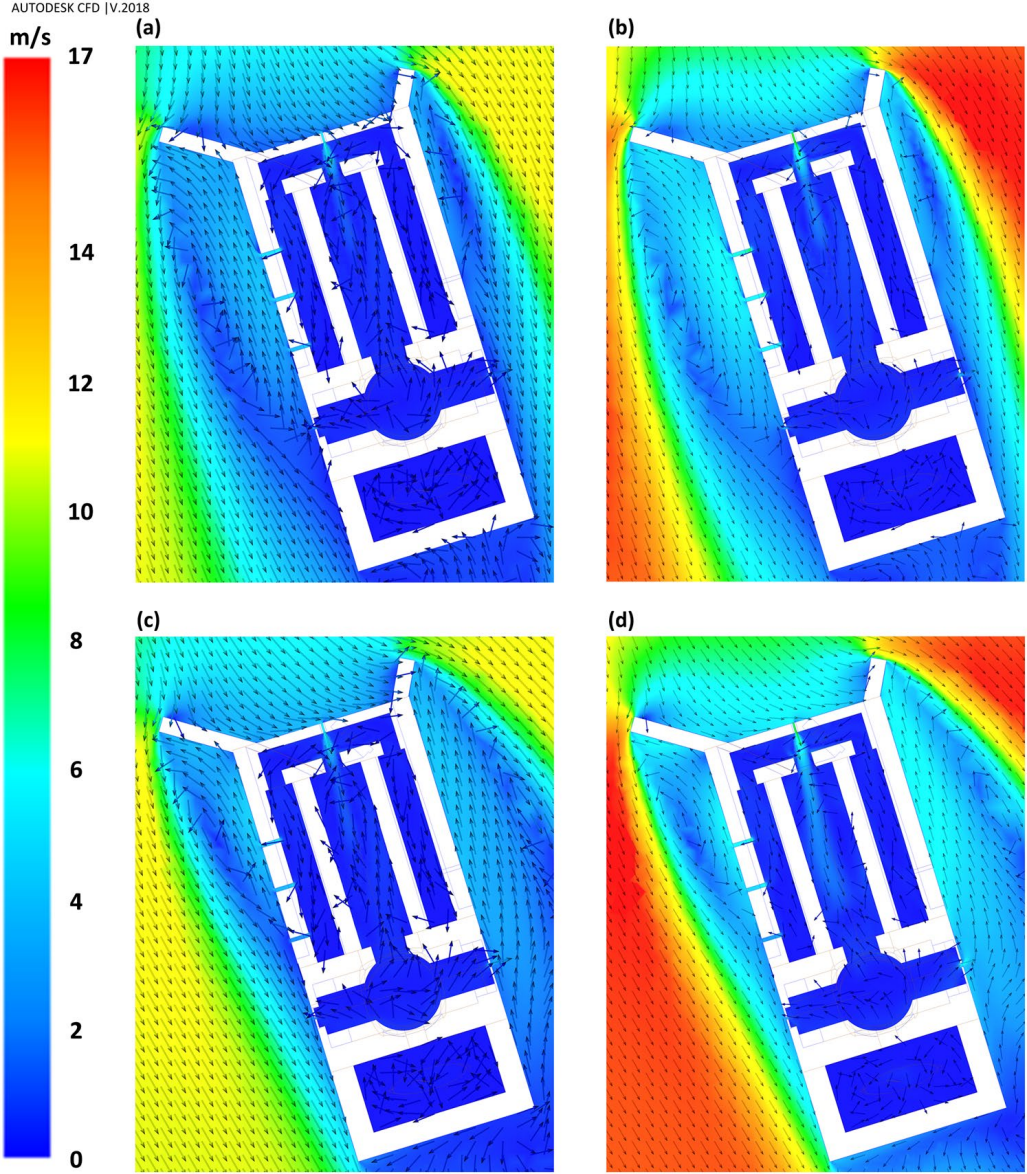
Flow pattern around the building in different scenarios: (**a**) Sc17-10, (**b**) Sc17-15, (**c**) Sc30-10, and (**d**) Sc30-15.

**Figure 13:**
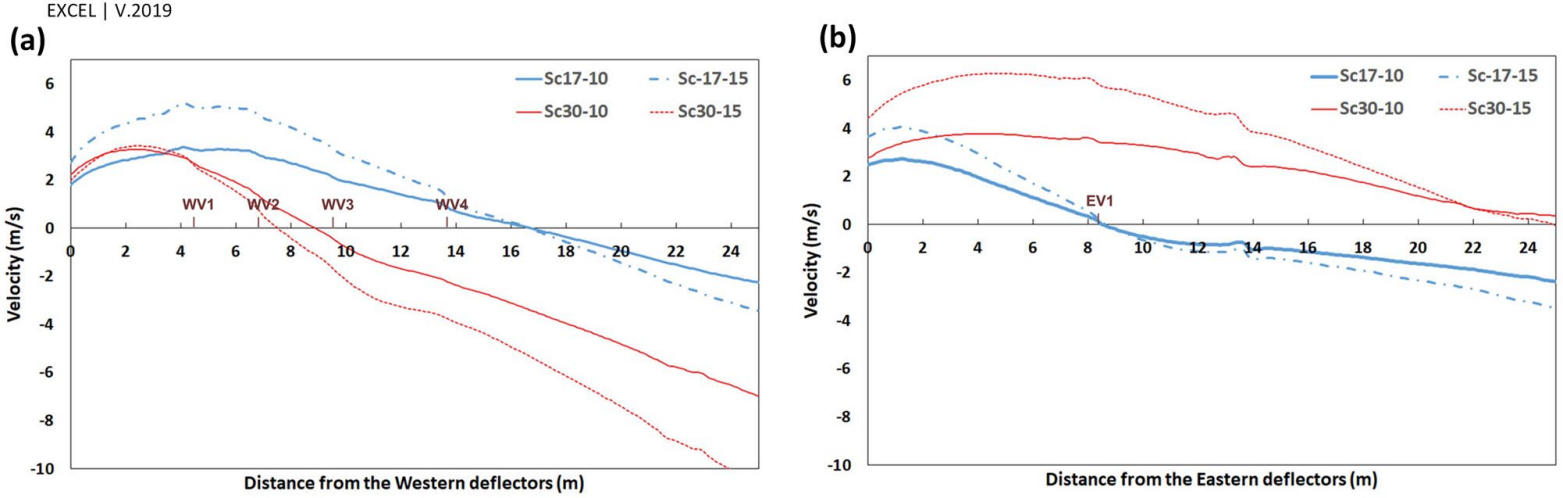
${V}_{y{^{\prime}}}$ on the profiles parallel and near the (**a**) western and (**b**) eastern wall, behind the deflectors.

**Figure 14:**
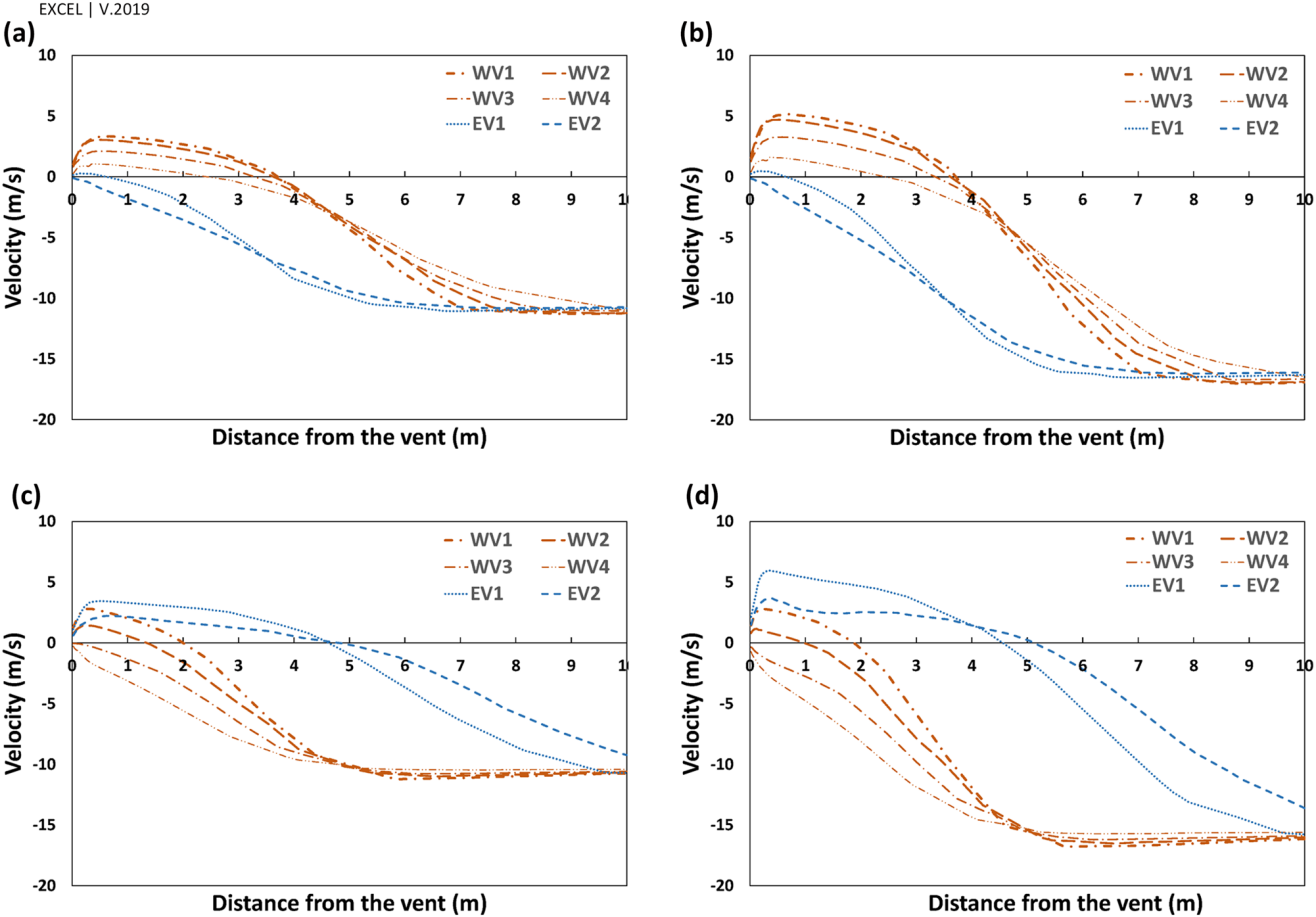
${\mathrm{V}}_{\mathrm{y{^{\prime}}}}$ on the perpendicular profiles to western and eastern vents in scenarios (**a**) Sc17-10, (**b**) Sc17-15, (**c**) Sc30-10 and (**d**) Sc30-15.

Flow patterns around the windmill have been depicted in Fig. 12. The ventilation by western and eastern vents was directly related to the vortices formed behind the deflectors (Figs. 12, 13, 14). In general, when the wind direction was $\omega =\beta =17^\circ$ (perpendicular to frontier wall with the angles of attack being ${\gamma }_{1}=55^\circ$ and ${\gamma }_{2}=29^\circ$), i.e., Sc17-10 and Sc17-15, the length of vortex behind western deflector was larger than behind the eastern one and vice versa, increasing the flow angle to $\omega =30^\circ$, i.e., Sc30-10 and Sc30-15 resulted in higher vortex length behind the eastern deflector.

The parallel component of flow to the walls, ${V}_{y{^{\prime}}}$, would be an appropriate estimation for the shape of currents around the windmill. The positive values, ${V}_{y{^{\prime}}}>0$, showed reverse flow, while the negative values illustrated current in the same direction as the dominant wind in the space (ordinary current). Thus, the wind speed (${V}_{y{^{\prime}}}$) profiles were considered to assess the vortices' length and width.

The vortices' length has been assessed by two 25-m profiles from deflectors near the eastern and western walls (Fig. 13).

Furthermore, the vortices' widths were measured by six profiles, ten meters away from the vents (Fig. 14). These graphs also demonstrated how the wind angles and speeds affected the vortices behind deflectors.

At $\omega =17^\circ$, the length of vortices was approximately 16.5 m and 8.5 m on the western and eastern wall, respectively, for both wind speeds, i.e., 10 and 15 m/s. Somehow, at $\omega =30^\circ ,$ the length of vortices was 7 m and 9 m for the wind speed of 10 and 15 m/s, respectively, on the western wall. This value occurred at more than 25 m behind the eastern deflector (higher than the structure length) at both wind speeds.

In general, the widths of vortices were more expansive on the western wall when the wind angle was $\omega =17^\circ$, while it was wider behind the eastern deflector at $\omega =30^\circ$ (Fig. 14). In these profiles, on the vortices, the maximum ${V}_{y{^{\prime}}}$ occurred approximately 50 cm away from the walls and then diminished upon increasing the distances from vents. At $\omega =17^\circ$, the flow currents were directed to a normal flow pattern at near 9 m on the western and 6.5 m on the eastern wall (Fig. 14). On the other hand, at $\omega =30^\circ$, they tended to normal flow pattern at 5 m and more than 10 m for western and eastern walls, respectively.

**Figure 15:**
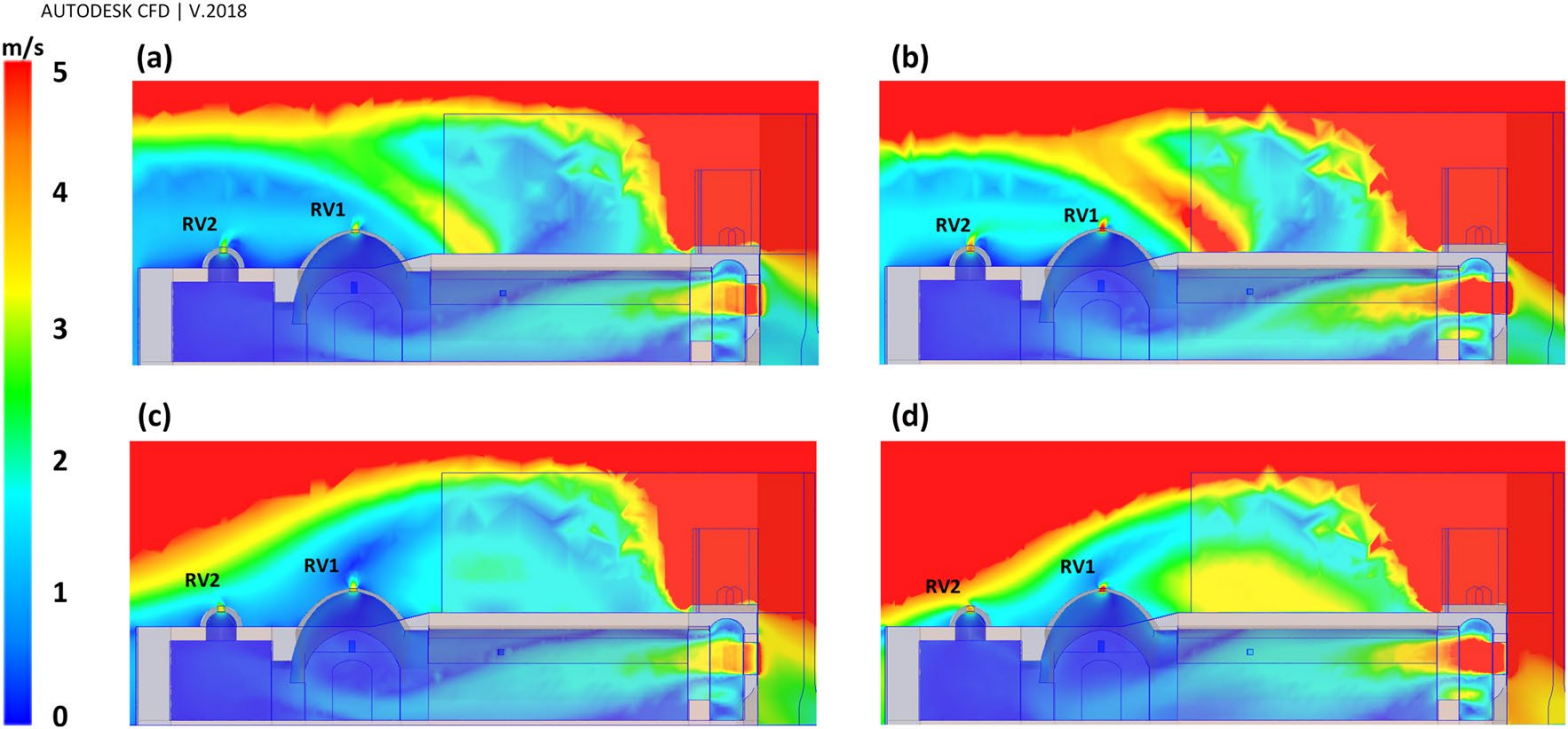
Flow pattern on the section A-A at different scenarios; (**a**) Sc17-10, (**b**) Sc17-15, (**c**) Sc30-10, and (**d**) Sc30-15.

In addition, the CFD results on sections A-A revealed that RV1 and RV2 contributed to natural ventilation properly (Fig. 15).

**Figure 16:**
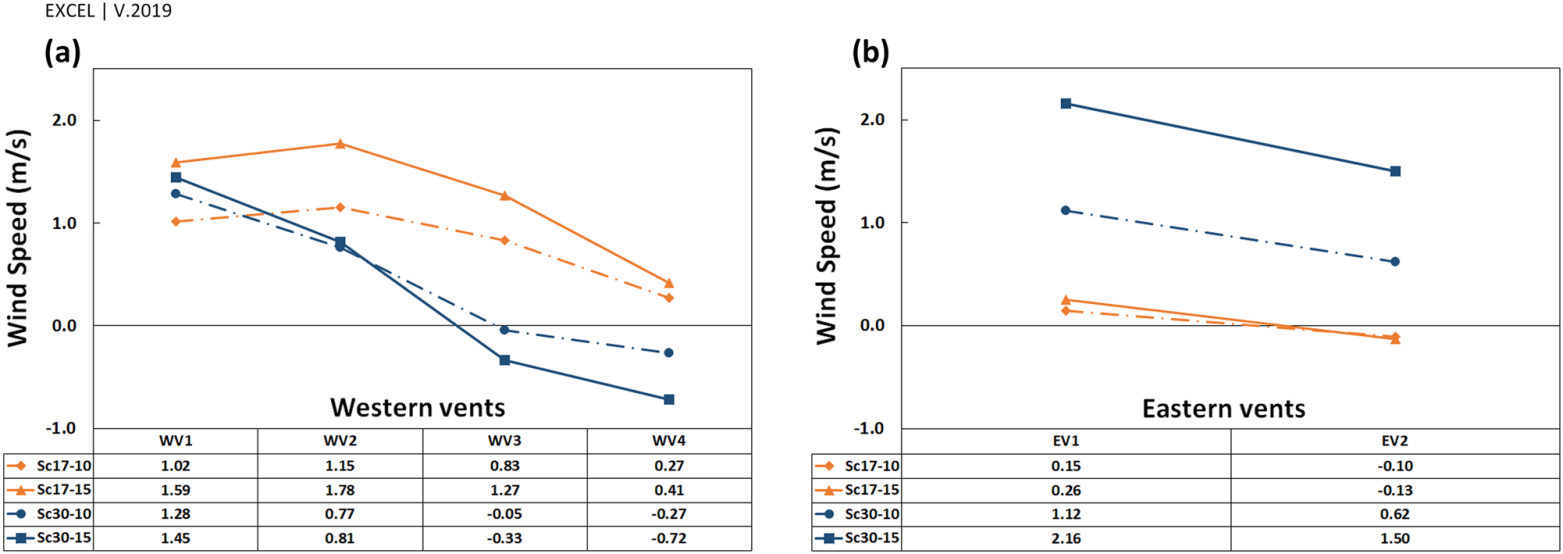
${V}_{y{^{\prime}}}$ of flow near the (**a**) western and (**b**) eastern vents.

**Figure 17:**
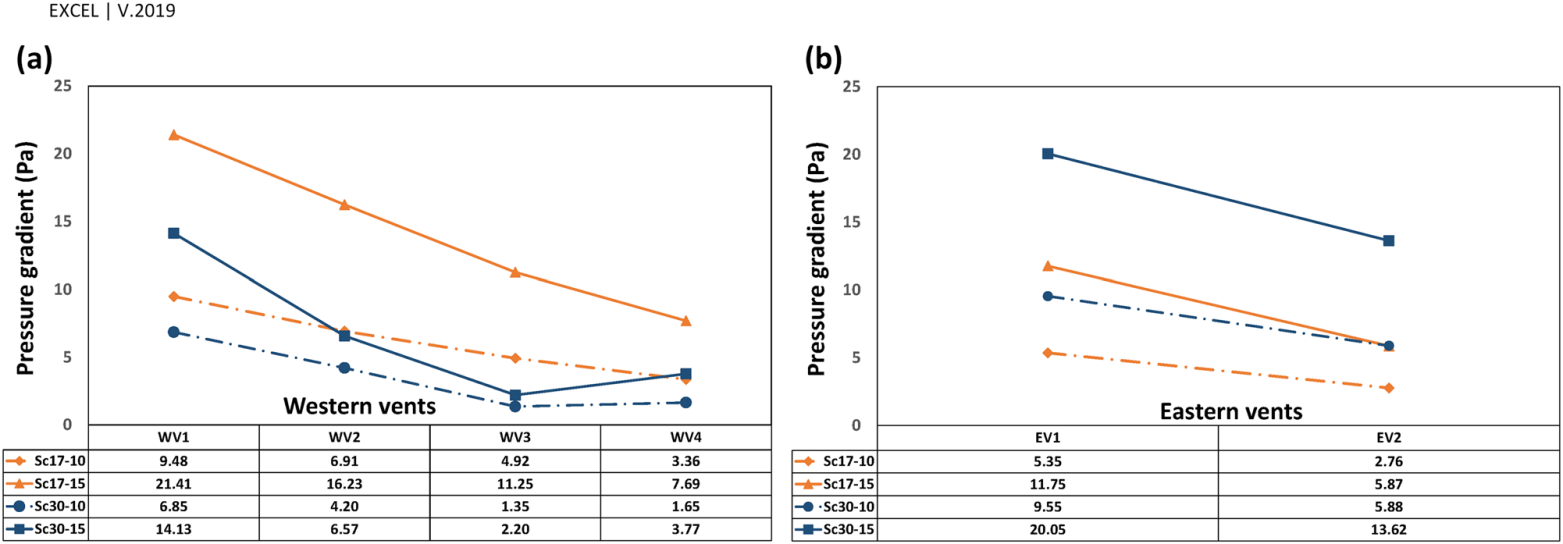
Pressure gradient in (**a**) western and (**b**) eastern vents.

The ${V}_{y\mathrm{^{\prime}}}$ near the western and eastern vents, and the pressure gradient in the vents has been depicted in Figs. 16 and 17, respectively. In general, the ventilation from vents was directly correlated with the wind current near the vents.

The pressure gradient in vents was generally reduced upon increasing the distance from deflectors. The trend differed in WV3 and WV4 at $\omega =30^\circ$ (Fig. 17a) as the absolute ${V}_{y\mathrm{^{\prime}}}$ was higher in WV4 than in WV3 (Fig. 16a).

When the wind angle was $\omega =30^\circ ,$ no swirl formed on WV3 and WV4, and the flow currents were normal flow patterns on these vents (Fig. 13). This is due to the formation length of vortices behind deflectors. At this angle, the vortex formed before WV3 (Fig. 12), after which the flow reached zero at a specified point before this vent and then increased steadily on WV3 and WV4. Thus, the absolute wind speed in WV4 was higher than in WV3 (Fig. 16). Further, when the wind angle was $\omega =17^\circ ,$ the vortices on eastern vents were small enough such that no vortices formed on EV2 (Fig. 13).

**Figure 18:**
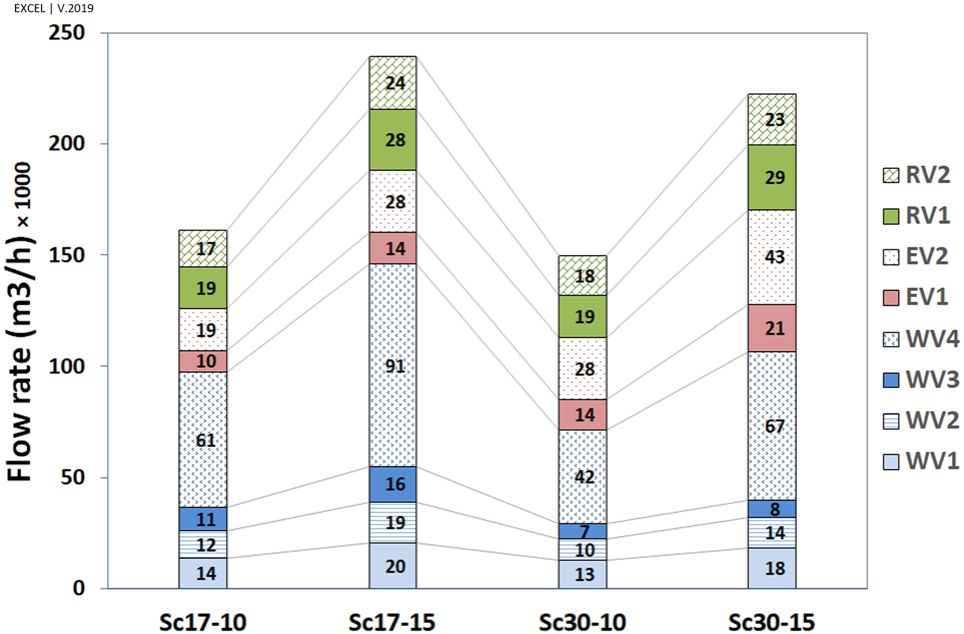
Contribution of different vents to ventilation of indoor.

The flow rate (FR, ${\mathrm{m}}^{3}/\mathrm{h})$ in vents has been calculated and depicted in Fig. 18. Apart from the flow pattern near the walls (reverse or ordinary), the flow rate was outside in all vents (i.e., vacuum). The results indicated that the maximum flow rate in all scenarios occurred in WV4 followed by EV2. Furthermore, the western vents would contribute to ventilation more than the eastern vents do, by 343% at the angle of $17^\circ$ and 169% at the angle of $30^\circ$, respectively.

The effect of windspeed on ventilation has been reported in Table 3. The results revealed that the flow rate has grown upon increasing wind speed from 10  to 15 m/s.

**Table 3:** Effect of wind speed on natural ventilation of the windmill.

Vents	Flow rate change (%) V15/V10 Wind direction = $17^\circ$	Flow rate change (%) V15/V10 Wind direction = $30^\circ$
EV1	151	154
EV2	148	154
WV1	151	146
WV2	151	136
WV3	151	118
WV4	148	158
RV1	146	152
RV2	145	130
Average	149	151

The effect of wind direction on ventilation has been outlined in Table 4. The results revealed that when the wind direction changed from $17^\circ$ to $30^\circ$, the ventilation increased dramatically in eastern vents (149% on average) and decreased in western vents (73% on average). The values dropped continually upon the increasing distance from the west deflector, except in WV4 because of different flow patterns on WV3 and WV4. The ventilation in roof vents did not change with the alteration of the wind direction.

**Table 4:** Effect of wind direction on natural ventilation of the windmill.

Vents	Flow rate change (%) $30^\circ /17^\circ$ V = 10 m/s	Flow rate change (%) $30^\circ /17^\circ$ V = 15 m/s
EV1	144	147
EV2	147	153
WV1	92	89
WV2	81	73
WV3	62	49
WV4	69	74
RV1	101	105
RV2	108	97
Average	93	93

**Figure 19:**
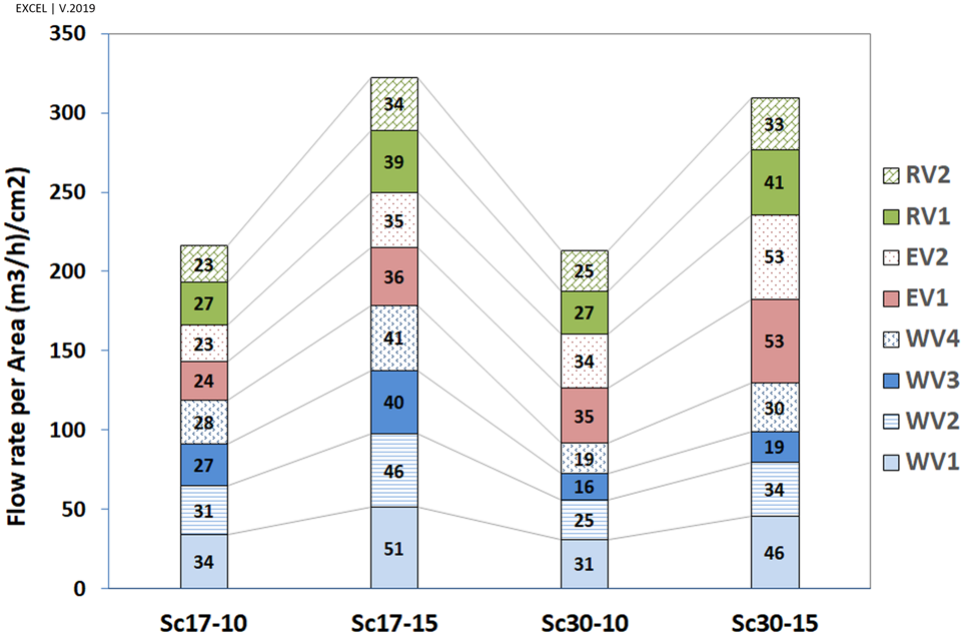
Average speed (Flow rate per area), ($\left({\mathrm{m}}^{3}/\mathrm{h}\right)/{\mathrm{cm}}^{2}$).

The higher flow rate in WV4 and EV2 was related to the higher surface area of these two vents. Accordingly, the average speed has been calculated, with the results illustrated in Fig. 19.

By looking at each scenario, when $\omega =17^\circ$, the highest and lowest average speeds occurred in WV1 and EV2, respectively. At $\omega =30^\circ$, the maximum and minimum average speeds took place in EV2 and WV3, respectively. Further, the lowest average speed occurred in WV3, followed by WV4 in the scenario of Sc30-10, as expected.

The analyses revealed that although the ventilation was higher on the western wall at $\omega =17^\circ$ by 126%, the eastern wall's contribution to ventilation was higher at $\omega =30^\circ$ (Table 5).

**Table 5:** The comparison of average speed in western vents (WVs) to eastern vents (EVs).

Scenarios	Sc17-10	Sc17-15	Sc30-10	Sc30-15
(1) Mean average speed in Western Vents	344	517	266	375
(2) Mean average speed in East Vents	274	410	399	616
The ratio of (1)/(2) (%)	126		64	

Further, corridor vents (C.V.) were more effective in natural ventilation than other vents (O.V.) (Table 6). This effect was highlighted in western vents. The maximum ratio of mean average speed in three western corridor vents (WCV) to WV4 occurred at Sc30-10 because of the lowest average speed value in WV4. Furthermore, as expected, the minimum value of WV3/WV4 occurred in Sc30-15.

**Table 6:** The average speed ratio in corridor vents to other vents.

	Sc17-10	Sc17-15	Sc30-10	Sc30-15
EV1/EV2	102	104	100	100
WCV/WV4	109	111	125	108
WV1/WV4	122	124	162	150
WV2/WV4	111	112	129	111
WV3/WV4	95	97	85	64

As stated earlier, near WV4, the flow pattern was normal in Sc30-15 and the absolute ${V}_{y{^{\prime}}}$ was higher than in Sc17-15 (on which the vortex has been formed), $\left| {\underbrace {{V_{{yWV4}}^{{'30 - 15}} }}_{{ - 0.72}}} \right| > \left| {\underbrace {{V_{{yWV4}}^{{'17 - 15}} }}_{{0.41}}} \right|$; however, the pressure gradient and average speed had a reverse pattern, $\underbrace {{\Delta P_{{WV4}}^{{30 - 15}} }}_{{3.77}} < \underbrace {{\Delta P_{{WV4}}^{{17 - 15}} }}_{{7.69}}$ and $\underbrace {{FRPA_{{WV4}}^{{30 - 15}} }}_{{352}} < \underbrace {{FRPA_{{WV4}}^{{17 - 15}} }}_{{477}}$.

Hence, it could be concluded that the flow in vortices would affect ventilation more than ordinary currents.

Looking at Fig. 16, near the vents ${V}_{y{^{\prime}}}$ had the maximum rate on the west in Sc17-15 rather than in other scenarios, leading to the highest-pressure gradient and average speed. In addition, in Sc30-10 and Sc30-15, the pressure gradient and average speed changed with ${V}_{y{^{\prime}}}$ (Table 7).

**Table 7:** Relation of $\left|{V}_{y{^{\prime}}}\right|$ near the vents, $\Delta P$ and average speed in Sc30-10 and Sc30-15.

	WV1		WV2		WV3		WV4
**Sc30-10**							
$\left|{V}_{y{^{\prime}}}\right|$	$\left|1.28\right|$	** > **	$\left|0.77\right|$	** > **	$\left|-0.05\right|$	** < **	$\left|-0.27\right|$
$\Delta P$	6.85	** > **	4.2	** > **	1.35	** < **	1.65
Average speed	362	** > **	287	** > **	190	** < **	223
**Sc30-15**							
$\left|{V}_{y{^{\prime}}}\right|$	$\left|1.45\right|$	** > **	$\left|0.81\right|$	** > **	$\left|-0.33\right|$	** < **	$\left|-0.72\right|$
$\Delta P$	14.13	** > **	6.57	** > **	2.20	** < **	3.77
Average speed	530	** > **	392	** > **	225	** < **	352

**Figure 20:**
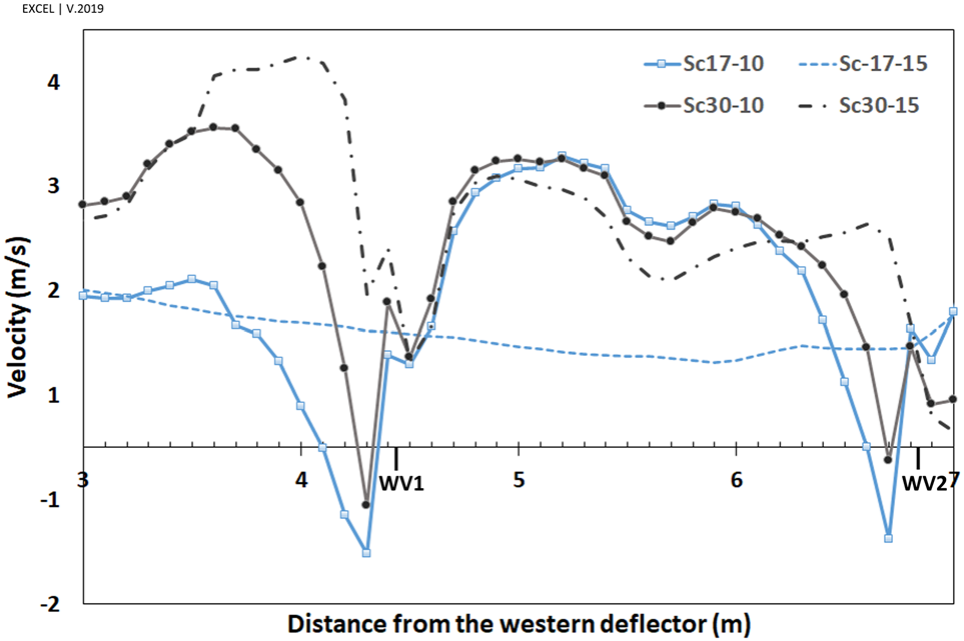
Microscale eddies near WV1 and WV2 behind the western deflector.

**Figure 21:**
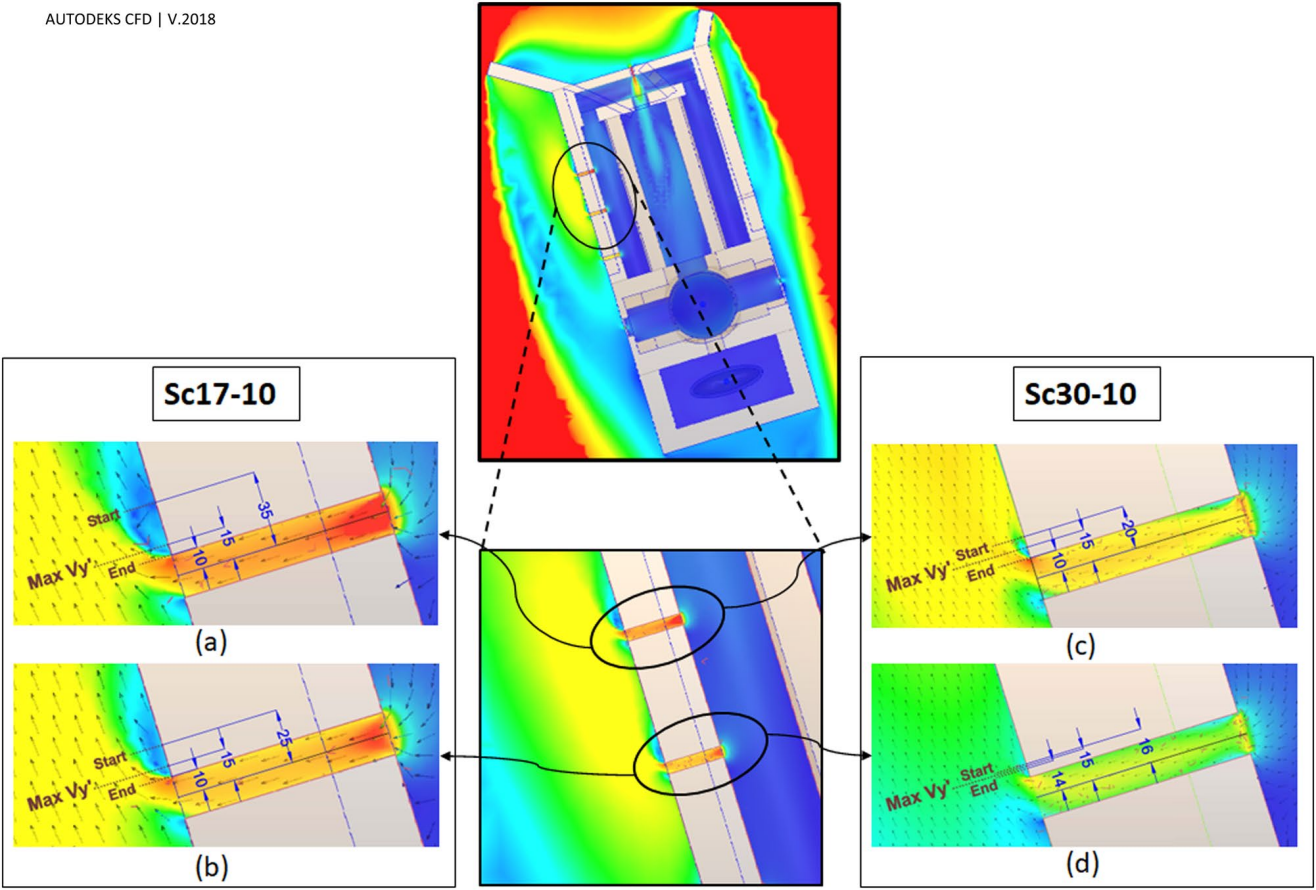
Eddies near (**a**) WV1 and (**b**) WV2 in Sc17-10 as well as (**c**) WV1 and (**d**) WV2 in Sc30-10.

This pattern was different in WV1 and WV2 in Sc17-10. In this scenario, although the average speed in WV2 was lower than in WV1 ($\underbrace {{FRPA_{{WV2}}^{{17 - 10}} }}_{{355}} < \underbrace {{FRPA_{{WV1}}^{{17 - 10}} }}_{{393}}$), ${V}_{y{^{\prime}}}$ near WV2 was higher than near WV1 ($\underbrace {{V_{{{y'}} _{{WV2}}}^{{17 - 10}} }}_{{1.15~{\text{m}}/{\text{s}}}} > \underbrace {{V_{{{y'}} _{{WV1}}}^{{17 - 10}} }}_{{1.02~{\text{m}}/{\text{s}}}}$). This could be due to microscale eddies formed near WV1 and WV2, while the reverse current in microscale eddy near WV1 was stronger than near WV2 (Figs. 20, 21). These microscale eddies formed just when the wind speed was 10 m/s.

#### Microscale eddy

At ${V}_{\infty }=10$m/s, if the wind angle was $\omega =17^\circ$, the micro eddy started at 35 cm away from the center of the WV1 (Fig. 21) and continued by 25 cm. In this eddy, the maximum absolute value of ${V}_{y{^{\prime}}}$ was $\left|{{V}_{y{^{\prime}}}}_{WV1}^{Sc17-10}\right|=1.02 \mathrm{m}/\mathrm{s}$, which occurred at 15 cm away from the center of the vent. In addition, the eddy was started from 25 cm to near 10 cm from the center of WV2 (the length was 15 cm), and the maximum absolute value was $\left|{{V}_{y{^{\prime}}}}_{WV2}^{Sc17-10}\right|=0.13 \mathrm{m}/\mathrm{s}$, occurring at a distance of 15 cm. When ${V}_{\infty }=10 \mathrm{m}/\mathrm{s}$ and $\omega =30^\circ$, the eddy formed from 20 to 10 cm from WV1 (the length was 10 cm) where the absolute maximum value was $\left|{{V}_{y{^{\prime}}}}_{WV1}^{Sc30-10}\right|=0.56 \mathrm{m}/\mathrm{s}$. Finally, in the same scenario and near WV2, the eddy formed 16 cm to 14 cm from the center of the vent (2 cm length) with the maximum value of $\left|{{V}_{y{^{\prime}}}}_{WV2}^{Sc30-10}\right|=0.13 \mathrm{m}/\mathrm{s}$ 15 cm away from the vent center.

Thus, as the maximum reverse flow occurred at the eddy in Sc17-10 near WV1, ${V}_{y{^{\prime}}}$ has diminished more near this vent in this scenario than in others (Fig. 16).

In addition, according to Fig. 16, the minimum ${V}_{y{^{\prime}}}$ in WV1 occurred in Sc17-10, resulting from the described micro-scale eddy.

### Thermal comfort and indoor air quality

The results indicate that the mean value of indoor air velocity in the four considered scenarios was about 0.31 m/s. On the other hand, in naturally ventilated buildings, the occupants’ thermal comfort must be evaluated based on adaptive thermal comfort models. As such, in the present study, we use the adaptive thermal comfort model of ASHRAE standard 55-2020^72^. Thus, by considering the summer outdoor mean temperature of the Sistan region (about 33 °C) and mean air velocity of 0.31 m/s, the adaptive comfort model reveals that without using any mechanical cooling systems for the indoor space of the windmill, the occupants’ thermal perception lies within the comfort range with 80% acceptability limit.

IAQ depends on various parameters such as air flow rate, air change rate, and local mean age of air (LMA) in a 1.5 m horizontal plane (level of standing human in activities). Since the air change per hour (ACH) parameter was extensively used in similar studies, this criterion has been utilized for evaluating the IAQ.

#### Air change per area

The air change per hour, ACH (/h), was calculated through dividing the integrated vent velocities by the room volume, V (Eq. 8)

8 $$ACH=3600.\frac{\int \overrightarrow{v}.dA=\frac{1}{2}\sum_{k}\sum_{j=1}^{n}\left|{v}_{j}.{n}_{j}\right|.{A}_{j}}{V}$$

where ${v}_{j}$ is velocity vector, ${n}_{j}$ is the ordinary vector to the vents surface, ${A}_{j}$ represents the area of jth cell, n is the total number of cells at the vents, and k shows the number of vents in the building.

**Figure 22:**
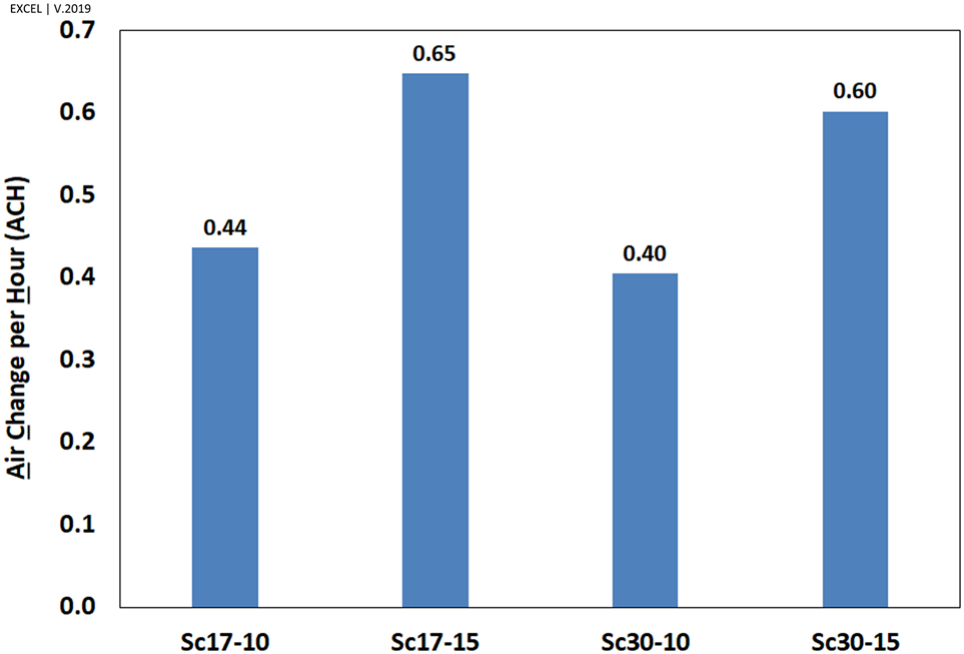
Air change per hour (ACH) in the windmill in different scenarios.

The results (Fig. 22) showed that all scenarios were beyond the ASHRAE standard for residential buildings (ACH = 0.35)^73,74^. This figure indicates that air change increases when the wind blows faster. One of the interesting findings is that the air change diminished by increasing the wind angle.

### Application in modern architecture

The application of wind in the ventilation of vernacular buildings was explained in various literature. Indeed, the important issue is how we can enhance the ventilation rate in modern architecture with this knowledge. Projects such as Kidderminster College^75^, and Windcatcher Zero^76^ are the cases that utilize natural ventilation in modern architecture^7,36^. The characteristics of the studied structure, including its orientation, the use of deflectors, and the shape and placement of vents as well as corridors can be a suitable model for harnessing the wind in indoor air exchange. Further, each of the mentioned approaches (such as the effect of negative pressure^43^ behind deflectors) or a combination of these approaches can be proposed as an effective solution for cross or single-side ventilation strategies. For example, this system could be recommended as a suitable auxiliary solution for the proper ventilation process in large public spaces such as halls, museums^77^, warehouses, enclosed stadiums^78^, and factories, which on the one hand, overcrowding increases the indoor-induce pollutants^79^ in the space, and on the other hand, the cost of ventilation in these places is high.

## Conclusion

This paper assessed wind-driven ventilation in an ancient structure, a windmill in Sistan, Iran. CFD analyses were applied to study the natural ventilation and validated by site measurement. The Sistan wind flows in the NW–SE direction by the resultant vector of $17^\circ$, fluctuating from 0° to 60°. Accordingly, the windmill was built based on the resultant wind vector in the region. Two deflectors were constructed wide enough to collect all winds within the mentioned range to utilize the wind in the higher range. Two western and eastern corridors were built to enhance ventilation.

This study chose four different climatic scenarios according to the Sistan wind pattern, i.e., two significant wind speeds by two prevailing wind directions. The current approach illustrated deflectors' functions in the formation of vortices and the contribution of vents to indoor ventilation. An error of 17% was calculated for validation because of the transient condition in the natural wind pattern and the high uncertainty in site measurements. The results revealed that the functionality of vents on the eastern and western walls would change dramatically with changes in wind speed and direction. In general, the findings of this study are summarized as follows:

1. Regarding natural ventilation, the deflectors had two main functions:
2. The most important function was to accumulate and direct the wind toward the wind entrance.
3. The second function involved the diminution of turbulent flow (by forming vortices) on the eastern and western walls to enhance indoor ventilation.
4. According to the meteorological data, the wind resultant vector in Sistan is $17^\circ$. In addition, regarding natural ventilation at $\omega =17^\circ$, the west wall contributed more than the east wall by 126%. Thus, more vents were designed and constructed on the western wall to enhance ventilation.
5. By changing the wind direction from $17^\circ$ to $30^\circ$, the more considerable vortex length behind eastern deflectors led to higher ventilation by 149% on this wall.
6. Although the higher wind speed (15 m/s) enhanced the ventilation in roof vents, the wind direction did not affect this value in RV1 and RV2.
7. The microscale eddy near WV1 led to a reduction in ${V}_{y{^{\prime}}}$ on this vent, especially in Sc17-10. It suggests designing a vent with a rounded edge to reduce the flow loss coefficient near vents^80^.
8. When the wind speed increased from 10 m/s to 15 m/s, the ACHs dropped by 150%. Further, by changing the wind angle from $17^\circ$ to $30^\circ$, the ACH dropped by 10%. However, the ACHs were higher than the ASHRAE standard (ACH > 0.35).

Some aspects should be specified to utilize wind in residential regions, including the current dynamic around buildings, the angle and speed of wind hitting the building, low and high-pressure areas, and vortices around the buildings. In this way, the complete flow pattern around the buildings could be determined and provided for both engineers and architects.

It is necessary to consider some limitations in this paper, such as the deflectors' geometrical characteristics (i.e., length and angle) on vacuum rate. The human comfort analyses (i.e., thermal, humidity, and wind speed effects) should be performed in further investigations considering the effect of corridors on preventing turbulent indoor flow. The impact of moist and porous media on the entrance (Kharkhona^53^ in the native language of Sistan) should be analyzed on the ENTRANCE.

The underlying principles uncovered by this research and the design type of this structure can be adopted in many buildings and enclosed spaces with a high rate of indoor-induced pollution. However, it is worth noting that not all strategies in designing this structure apply to every location and it is recommended to use this under similar climatic conditions.

## Data Availability

The author would like to thank the Global Wind Atlas for access to the wind map of Sistan. The datasets analyzed during the current study are available in the figshare repository, 10.6084/m9.figshare.19775980.
